# Trunk isometric force production parameters during *erector spinae* muscle vibration at different frequencies

**DOI:** 10.1186/1743-0003-10-89

**Published:** 2013-08-06

**Authors:** Jean-Alexandre Boucher, Martin C Normand, Martin Descarreaux

**Affiliations:** 1 Département de psychologie, Université du Québec à Trois-Rivières, 3351 Boul. des Forges, C.P. 500, G9A 5H7, Trois-Rivières, QC, Canada; 2 Département de chiropratique, Université du Québec à Trois-Rivières, 3351 Boul. des Forges, C.P. 500, G9A 5H7, Trois-Rivières, QC, Canada

**Keywords:** Muscle vibration, Muscle spindle, Low back, Neuromuscular responses, Isometric force, Proprioception, *Erector spinae* muscles

## Abstract

**Background:**

Vibration is known to alter proprioceptive afferents and create a tonic vibration reflex. The control of force and its variability are often considered determinants of motor performance and neuromuscular control. However, the effect of vibration on paraspinal muscle control and force production remains to be determined.

**Methods:**

Twenty-one healthy adults were asked to perform isometric trunk flexion and extension torque at 60% of their maximal voluntary isometric contraction, under three different vibration conditions: no vibration, vibration frequencies of 30 Hz and 80 Hz. Eighteen isometric contractions were performed under each condition without any feedback. Mechanical vibrations were applied bilaterally over the lumbar *erector spinae* muscles while participants were in neutral standing position. Time to peak torque (TPT), variable error (VE) as well as constant error (CE) and absolute error (AE) in peak torque were calculated and compared between conditions.

**Results:**

The main finding suggests that *erector spinae* muscle vibration significantly decreases the accuracy in a trunk extension isometric force reproduction task. There was no difference between both vibration frequencies with regard to force production parameters. Antagonist muscles do not seem to be directly affected by vibration stimulation when performing a trunk isometric task.

**Conclusions:**

The results suggest that acute *erector spinae* muscle vibration interferes with torque generation sequence of the trunk by distorting proprioceptive information in healthy participants.

## Background

The study of acute vibration effect on muscle force, power, balance and proprioception parameters is gaining popularity in the field of exercise physiology and physical rehabilitation. Over the past decades, numerous publications using non-standardized protocols tried to identify the underlying neural mechanisms responsible for the effects of acute vibration. As reported by a recent Cochrane review [1], such neural mechanisms remain equivocal due to the large number of studies using different methods of vibration application, vibration parameters and exercise regimens. One can therefore argue that different vibration frequencies, amplitudes and durations can potentially influence the outcome measures, making it difficult to compare the various study results. However, the local neurophysiological responses of a muscle to isolated vibration remain very well studied. Most authors seem to agree with the hypothesis that mechanical vibration tends to create a rapid lengthening and shortening phase of the vibrated muscle [2,3], resulting in a phase-oriented discharge mainly from primary endings [4-7], but also from secondary endings [4,5,8]. It has been reported that while intrafusal fibers are stretched, the sensory endings are also stretched and consequently increase their firing rate. This discharge generates an excitatory response via a monosynaptic pathway involving the motor innervation from the large-diameter alpha motor neurons. As reported by Granit *et al*. [9], secondary endings were found to elicit a similar excitatory response through a polysynaptic pathway, and thus foster contractions of the homonymous muscle. Burke *et al*. [10] and Hayward *et al*. [11] have proposed that Ib-afferents from Golgi tendon organs are likewise reactive to muscle vibration, becoming more and more responsive when the muscle is contracting. It is also suggested that a vibration stimulus is capable of increasing muscle spindle activity, causing an excitatory response in the primary endings of a non-contracting muscle [10,12]. An ensuing contraction of the vibrated muscle, combined with inhibition of its antagonists, yields a tonic vibration reflex (TVR) [13-15].

Sustained muscle vibration is believed to introduce a bias that distorts the output of the Ia-afferents originating from the muscle spindles. The vibrated muscle is usually perceived to be longer than it actually is [16]. It has also been shown that primary and secondary endings respond in a submaximal manner when the muscle is in a relaxed state prior to contraction. While performing isometric voluntary contractions, the response of muscle spindle endings to vibration is maintained or increased under certain conditions as it seems to be attributed to the co-activation of the fusimotor system. Besides spinal reflex mechanisms, there is recent evidence suggesting that muscle tuning components and central motor command contribution also play a preponderant role in motor response to acute musculotendinous vibration [17,18]. It has also been reported that Ia afferent input has the ability to excite the corticospinal pathways [19] and activate the cortical motor areas [20].

In order to evaluate motor behavior during vibration exposure, several authors have used repositioning task protocols involving either upper or lower limb muscles. Studies conducted by Capaday and Cooke [21], Cody *et al*. [22] and Kasai *et al*. [23] have shown that muscle vibration distorts the perception of static joint angle and movement causing systematic errors in the end point of movement. To date, few studies investigated the effects of vibration on trunk repositioning task performances. Fontana *et al*. [24] concluded that an exercise regimen including weightbearing exercises under low frequency whole body vibration may lead to improvements in lumbosacral repositioning accuracy. Alternatively, Brumagne *et al*. [25] suggested that muscle vibration applied at segmental level L5-S1 leads to significant increase in pelvis directional error in a sitting position as illustrated by a systematic undershooting of the target position. The authors concluded that further research on the effect of vibration on healthy individuals in other postures and other muscle groups was desirable to elucidate the complex mechanism of lumbosacral neuromuscular function.

The control of force production and its variability are often considered the principal factors of several motor control models aiming at the understanding of skillful behaviors. To our knowledge, trunk isometric force production parameters and their variability have not been studied (in a motor control context) under conditions of *erector spinae* muscle vibration. While attempting to produce a given target force repeatedly, the initial impulse for producing the force can be linked to the neuromuscular activity necessary to produce the action [26]. The use of isometric contractions to assess force production parameters in a repositioning task has been suggested to reflect various limitations of the neuromuscular system [27].

Therefore, the objective of this study was to determine whether or not the application of vibration alters the control of trunk isometric force production. The results of this study will help clarify the mechanisms explaining the role of vibration in the improvement or the disruption of sensorimotor control related to *erector spinae* muscles, while specifying the vibration parameters most likely to create the desired changes. Such information is relevant to the broader question of how muscle spindles signal spine force production during trunk isometric contractions under vibration influence. The authors tested the hypothesis that *erector spinae* muscle vibration disrupts motor control, making it less accurate and more inconsistent to perform an isometric force reproduction task.

## Methods

### Participants

Force production parameters were measured in 21 healthy participants, 11 males and 10 females, with no history of chronic or recurrent low back pain, ranging in age from 19 to 54 years (age, 24.3 ± 7.6 years; height, 172.3 ± 7.9 cm; weight, 69.4 ± 12.6 kg). All volunteers were recruited from the university population. Ethical approval for the study was granted by the university local ethics committee. Exclusion criteria were any acute/chronic thoracic or low back pain history, ankylosing spondylitis, trunk neuromuscular disease, inflammatory arthritis, scoliosis (15° or more) and previous spinal surgery. Before testing, each participant was informed of the procedures and gave their written consent. In order to assess occupational physical activity and sports during leisure time, participants also completed the Baecke-f questionnaire [28]. This questionnaire was filled out to ensure that the physical activity levels did not differ between participants. Basic data on study participants are shown in Table 1.

**Table 1:** Basic data on study participants

**Parameter**	
*N*	21
Age, yr	24.3 ± 7.6
Weight, kg	69.4 ± 12.6
Height, cm	172.3 ± 7.9
Baecke-f questionnaire	
simple sports score	5.9 ± 3.5
leisure index	3.1 ± 0.5

### Preparatory procedures

**Figure 1:**
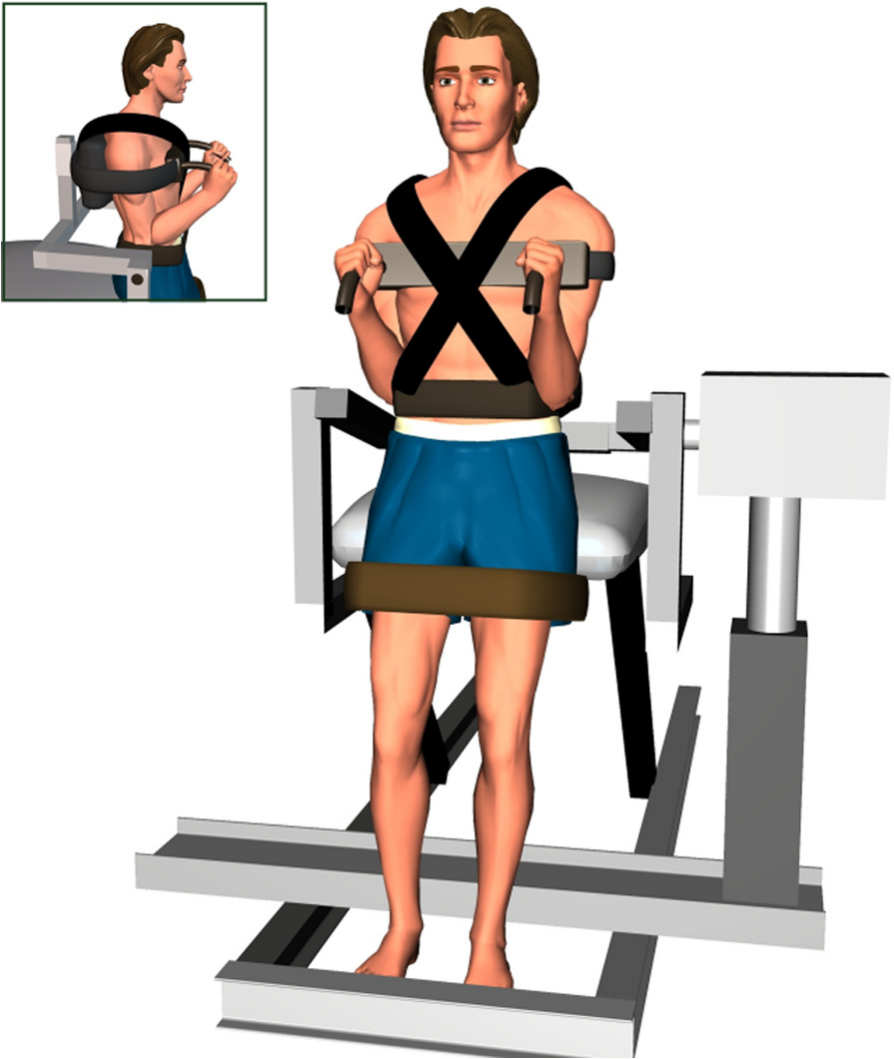
Testing position in neutral standing posture with and without mechanical vibration.

Testing was performed in a neutral standing posture (no trunk flexion or extension) with the set-up shown in Figure 1. Force data (torque) was obtained from an isokinetic device (The LIDO Active, Loredan Biomedical, West Sacramento, USA) used only in the isometric testing mode. First, maximal isometric flexion and extension torques of trunk muscles were collected while participants received personal encouragements from the experimenters. The higher torque value obtained in two consecutive 4-seconds trials was used as the reference for maximal voluntary contraction (MVC). After establishing the MVC, participants were instructed to produce a sub-maximal trunk isometric force as quickly as possible as a warm-up procedure for each condition (flexion and extension). For the learning phase, they were told to produce a single impulse ("shoot and release") and to make no attempt at correcting the force once the contraction was initiated. During this phase, participants were given visual accuracy feedback through an oscilloscope located in front of them. They were able to evaluate their performance and correct it for the next trial, if necessary. Participants were specifically asked to produce peak torques that were within 10% of the target goal set at 60% of their MVC, while keeping their eyes open for the entire session. The learning phase, completed without any form of vibration, was stopped when ten contractions were performed. This procedure was used to ensure that all participants understood and adequately performed the experimental task. For every trial, torque data were recorded at a sampling frequency of 100 Hz. They were digitally filtered with an eighth-order Butterworth filter (10 Hz low-pass cut-off frequency).

### Muscle vibration protocol

**Figure 2:**
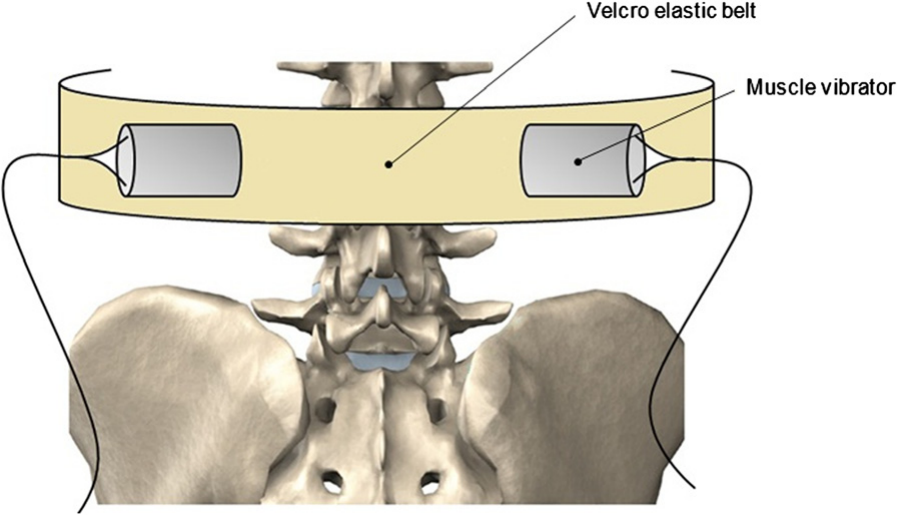
Experimental set-up for location of the applied vibration.

Superficial mechanical vibration was applied perpendicularly and bilaterally on lumbar *erector spinae* muscles at the third lumbar segment level (L3). Vibrators designed with a regulated DC power supply (Zurich, RPS-1012 MB) were held in place with a custom-made Velcro elastic lumbar belt (see Figure 2). The vibrators were placed in a standard position on all participants, by the same examiner, to ensure that the belt was secured with the same tension in all tests. This guaranteed the consistency of the applied vibration. Vibration frequencies used were 30 and 80 Hz with constant amplitude of 0.85 mm. These vibration characteristics were chosen in agreement with those suggested by Cardinale & Lim [29], who found an increased muscle activity when the vibration frequency was set at 30 Hz during whole body vibration, as well as Roll *et al*. [30] and Calvin-Figuière *et al*. [31] who suggested that 80 Hz vibration induced optimal kinesthetic illusions.

**Figure 3:**
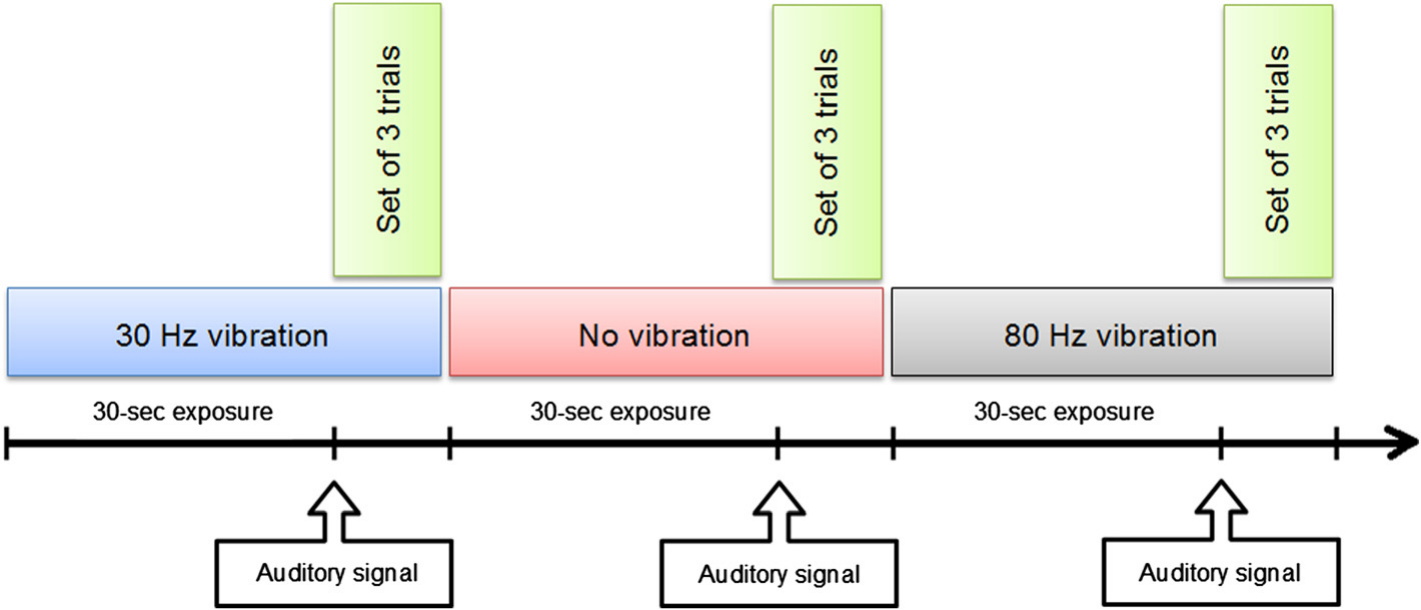
**Standard timeline of one block of trials.** The sequence in which each block of trials was presented was counterbalanced across participants.

**Figure 4:**
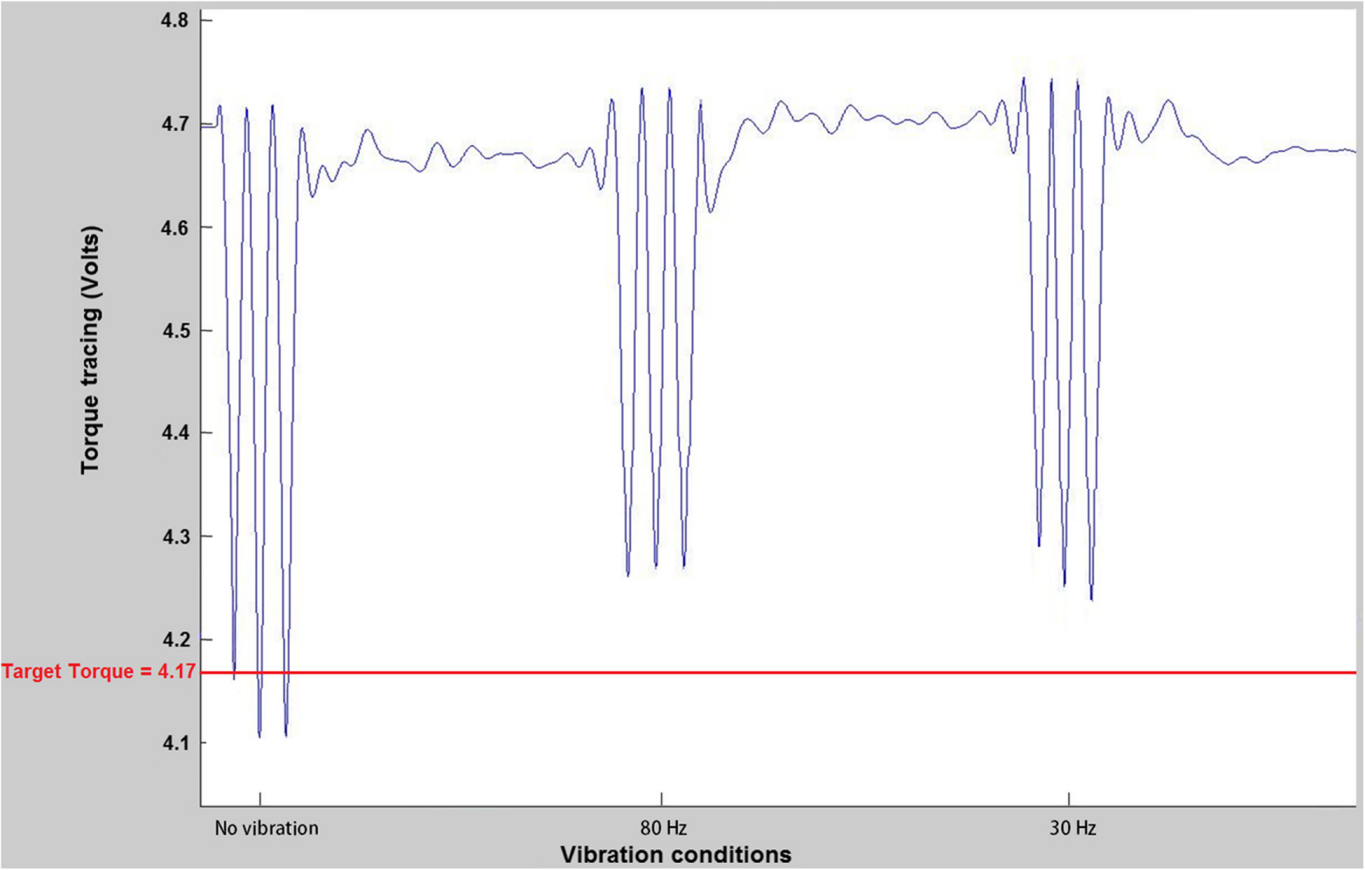
Example of one participant’s peak torque tracings in trunk extension representing one block.

Flexion condition was tested first, and no visual or verbal feedbacks were provided during experimental trials. Participants were asked to perform a set of three trials following an auditory signal which was heard every thirty seconds, for each of the vibration conditions (no vibration, 30 Hz and 80 Hz). That sequence represented one block of trials (see Figure 3). A total of three blocks were completed for each flexion and extension condition for a total of nine trials for each of the vibration condition. A 5-minute rest period between each block was allowed to limit possible sequence or fatigue effects. Vibrations were applied thirty seconds before each auditory signal, with the onset order being predetermined for each block, and lasted during the torque generation trials. The vibration, therefore, was applied without interruption throughout every vibration conditions without any rest or delay. The sequence in which each block of trials was presented was counterbalanced across participants. Figure 4 shows a representative example of peak torque tracings in trunk extension for the three vibration conditions, including the target torque.

### Data and statistical analyses

Time to peak torque (TPT), variable error (VE) as well as constant error (CE) and absolute error (AE) in peak torque were calculated and compared between vibration conditions in both flexion and extension. For each experimental trial, the onset of torque and peak torque were determined. Using this information, VE, CE and AE in peak torque were calculated for each condition. VE measures the inconsistency in movement outcome. It represents the difference between the participant’s peak torque score on each trial and his or her own average score. CE represents the positive or negative difference between the peak torque reached and the target torque corresponding to 60% of the MVC. A positive CE in trunk flexion corresponds to overshooting the target torque and a negative CE corresponds to undershooting the target torque. Inversely, a positive CE in trunk extension corresponds to undershooting the target torque and a negative CE corresponds to overshooting the target torque. AE in peak torque represents the average absolute deviation (without regard to torque direction) between the participant’s responses and the target torque [3]. A two-factor within-participants ANOVA design (3 vibration frequencies x 2 directions of exertion) was used for this study. The significance level was set at *P* < 0.05 for all analyses, and post-hoc comparisons, when needed, were conducted using the Bonferroni test.

## Results

**Figure 5:**
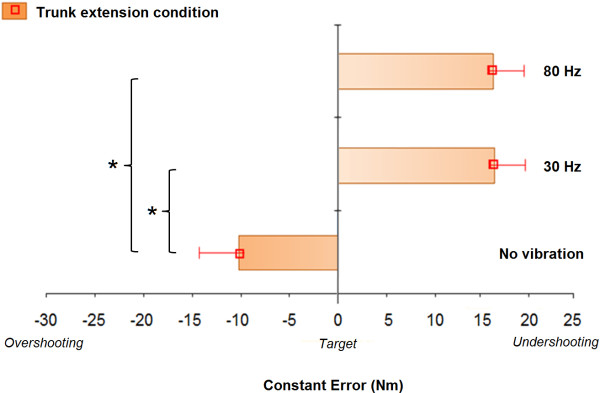
Comparison of mean constant errors in trunk extension task for each vibration condition: no vibration, 30 Hz vibration, and 80 Hz vibration (mean ± standard error).

The average MVC was 113.24 ± 58.11 Nm in trunk flexion and 128.06 ± 72.51 Nm in trunk extension. The statistical analysis yielded a significant difference in CE between the three vibration conditions in trunk extension (F(2,40) = 12.883, *P* < 0.001). Post-Hoc comparisons revealed significant increase in CE (undershoot) for both 30 Hz and 80 Hz vibration conditions (all *P* < 0.001) when compared to the no vibration condition. This observation is illustrated in Figure 5. However, 30 Hz and 80 Hz conditions were not different from one another (*P* = 1.00). The VE (F(2,40) = 0.034, *P* = 0.967) and AE (F(2,40) = 1.899, *P* = 0.163) values in trunk extension were not significantly different across conditions. On average, the TPT in trunk extension was 466.65 ± 8.49 ms and did not differ significantly between the three vibration conditions (*P* > 0.05). Table 2 displays the mean TPT, VE, CE, and AE scores for the three conditions in trunk extension. Statistical analyses for TPT, VE, CE and AE in trunk flexion yielded no significant difference (all *P* > 0.05). No significant vibration frequencies by directions of exertion interaction effect could be observed for the mean VE (F(2,40) = 0.198, *P* = 0.821), CE (F(2,40) = 14.556, *P* = 0.620) and AE scores (F(2,40) = 0.512, *P* = 0.603).

**Table 2:** Mean (±SD) time to peak torque (TPT) in ms, variable errors (VE), constant errors (CE), and absolute errors (AE) in Nm for the three vibration conditions in trunk extension

	**TPT**	**VE**	**CE**	**AE**
No vibration	462.74 ± 149.71	1.14 ± 1.53	-10.15 ± 4.75	9.21 ± 3.47
30 Hz vibration	476.39 ± 153.96	1.12 ± 1.38	16.36 ± 3.56	9.05 ± 3.19
80 Hz vibration	460.81 ± 137.47	1.15 ± 1.73	16.18 ± 3.65	9.28 ± 3.50

## Discussion

The aim of the present study was to determine whether or not the application of *erector spinae* muscle vibration alters the control of isometric force production of the trunk. The main findings suggest that *erector spinae* muscle vibration applied perpendicularly and bilaterally, at both 30 Hz and 80 Hz, significantly decreases the accuracy in a trunk extension isometric force reproduction task. Healthy participants were therefore less accurate in the extension force reproduction task corresponding to 60% of their MVC during *erector spinae* vibration and consequently undershot the target. The application of vibration, however, did not alter the torque trial consistency (VE) in any way. In a motor control context, CE and VE scores are preferable to AE values, as these error measurements can be interpreted more easily. However, CE scores do not consider the amount of scatter, variability, or inconsistency in performance of the torques [3]. Regarding the CE scores related to the no vibration condition, the question still remains as to why participants overshot the target torque. Hypothetically, one can believe the group of participants, in a general manner, tended to overshoot the target. However, even if they did so, the CE mean value remained lower when compared to the two vibration conditions. With this in mind, one can argue that there should be significant differences between the AE values in trunk extension. A controversy, however, exists about the use of AE. The mathematical properties of AE have been shown to be a complex combination of CE and VE, and it remains difficult to precisely assess the relative contribution of each measurement to AE [3]. The fact that TPT did not differ between the two vibration conditions suggests that participants did not modify their force production control strategy when vibration was applied. As suggested by Gordon and Ghez [26], when participants attempt to be as accurate as possible in a force reproduction task, they more consistently regulate force rise time around a constant value. In the present study, TPT was not expected to change, as instructions provided to participants prior to the experimentation focused essentially on precision.

Commonly reported muscle lengthening illusion in response to vibration has already been widely investigated [7,16]. Supported by repeated study observations, Eklund [32] found that muscle vibration can cause movement-illusions during isometric conditions. Kasai *et al*. [33], who studied the effect of vibration applied to postural muscles on anticipatory postural adjustment, reported about the central nervous system’s (CNS) ability to integrate proprioceptive messages arising from different muscles. They suggested on this basis that proprioceptive inputs might inject erroneous signals to the CNS and lead to distortion of the postural adjustment’s coordinate system. In their study conducted on dynamic sitting position sense of the lumbosacral spine, Brumagne *et al*. [25] also showed that vibration successfully induced a muscle lengthening illusion which led to a significant reduction in repositioning accuracy (increased CE under the vibration condition). Interestingly, in the present study isometric force reproduction task under *erector spinae* muscle vibration also led to an increased CE (reduced accuracy). However, it should be pointed out that fundamental differences are observed between dynamic and isometric movements. When performing an isometric contraction, there is a close relationship between impulse frequency in single spindle afferents and the strength of isometric contractions [34]. The average discharge of primary endings seems to remain constant and dependent on the torque generated by the isometric contraction [34]. Conversely, shortening contraction is well known to unload spindle endings; consequently, reducing muscle spindle firing rate endings seems to remain constant and dependent on the torque generated by the contraction [35]. Still, the hypothesis that vibration induces a kinesthetic illusion (in the direction that would produce stretching of the stimulated muscles) responsible for decreases in accuracy in this study should be considered. The positive CE found in trunk extension may correspond with an *erector spinae* lengthening illusion perceived by participants as they felt their trunk was leaning forward during vibration exposure. It is therefore possible that such lengthening illusion in trunk flexion has led participants to undershoot the target in trunk extension. Undershooting is consistent with an overestimate of required torque to achieve the target. In an interesting manner, Cafarelli and Kostka [36] found that vibration applied to the tendons during isometric contractions leads to an overestimation of the force generated by 30% and conversely, a 25% lesser than intended force is generated.

Burke *et al*. [5] were the first to study the effects of vibration on isometric voluntary contractions. The authors found that many of the spindle endings showed significant decreases in response to vibration with the appearance of the TVR. The discharge, however, remained locked to vibration cycles, and a partial recovery of the vibration responsiveness of primary endings occurred with prolonged vibration. Cordo *et al*. [37] also reported the timing of vibration being a key factor in the motor response. The methodological design of their study aimed at comparing three timings of vibration while performing a pointing task. If vibration began at the onset of movements, participants undershot the target. If vibration started 5 seconds before movement onset and continued throughout the movement, the undershoot error increased in magnitude. However, if vibration started 5 seconds before movement onset and then stopped at movement onset, participants overshot the target. Because participants, in our experiment, were exposed to 30 seconds bouts of vibration before torque onset and during torque trials, such a prolonged vibration exposure could somehow account for the positive CE found. In accordance with the timing principles investigated by Cordo *et al*., vibration exposure in this study could play some sort of role in increasing the magnitude of the errors (undershooting).

Physiological messages triggered by ongoing motor activities undergo a series of changes during the exposure of muscles to vibration [30]. From a neurophysiological point of view, acute muscle vibration may induce two types of muscle spindle adaptations: alterations in spindle sensitivity [38,39] or distortions in muscle primary afferents [10,16,40]. The question still arises on how different vibration frequencies with constant amplitude could lead or not to distinctive alterations in muscle spindle sensitivity or afferent distortions. A few authors attempted to compare various vibration frequencies in an exercise training perspective using whole body vibration [29,41] or in microneurographic studies [7,12,30]. These findings suggest vibration frequencies have distinctive muscle spindle primary ending discharge ratios. Although a greater kinesthetic illusion with 80 Hz was expected, no significant difference was observed with regard to vibration frequencies in the present study. This could be explained in part by acute vibration application on lower back muscles having several musculotendinous layers and degrees of freedom, which potentially differs from a vibration applied on a single monoarticular muscle.

Absence of significant results in trunk flexion is explained by the fact that vibration was applied on lumbar *erector spinae* muscles. These findings reinforce the main idea that mechanical vibration has a local influence on its vibrated muscles [39] and confirm that vibration of *erector spinae* muscles did not spread to surrounding structures through passive tissues or the custom-made Velcro elastic lumbar belt. Therefore, antagonist muscles do not seem to be directly affected by vibration stimulation when performing a trunk isometric task.

### Clinical applications

Vibration as a therapeutic modality is gaining in popularity. However, the clinical efficacy of vibration in the treatment of musculoskeletal disorders remains to be determined. Beneficial effect of vibration stimulation has been described in patients with non-specific low back pain (LBP). For instance, Brumagne et al. [42] reported increased trunk repositioning accuracy in patients with LBP submitted to paraspinal vibration. As previously mentioned, two hypotheses regarding muscle vibration effects have been suggested: muscle vibration could either distort muscle’s primary afferent by introducing a bias signal in a parallel channel [10,16,30,40], or have beneficial effects because of a stochastic resonance-based enhancement of proprioceptive acuity [38,43]. Results from laboratory studies usually have limited generalizability. Nevertheless, the present findings suggest that vibration can alter proprioceptive inputs from *erector spinae* muscles and could therefore be used as an additional challenge to the sensorimotor system during rehabilitation exercises.

### Study limitations

In accordance with Brumagne *et al*.’s [44] consideration about proprioceptive evaluation based on position and movement sense, a possible limitation of this form of evaluation is the fact that pointing task assessments stem from conscious control and memory, while proprioception control in the overall scheme of things is a more sub-conscious process. The experimental design of this study was not to provide any new evidence on the contribution of central pathways aiming at identifying the complete proper neural mechanisms, but to evaluate the motor response to acute local vibration exposure. Further vibration studies conducted on lumbar spine would benefit from monitoring motor unit and neural conduction activity in a sensorimotor control perspective.

## Conclusions

On the basis of the results presented in this study as well as previously reported findings on repositioning task protocols, it seems that acute *erector spinae* muscle vibration interferes with torque generation sequence by distorting proprioceptive information resulting in muscle lengthening illusion. The current study provides evidence that precise muscle spindle input of *erector spinae* muscles is crucial for accurate spine isometric force production in a neutral standing position. It is important to note, however, that paraspinal musculature has several musculotendinous layers, so further studies should be done on a mono-articular muscle to validate the results of this particular research before conducting them on low back pain populations.

## Abbreviations

AE: Absolute error; ANOVA: Analysis of variance; CE: Constant error; CNS: Central nervous system; LBP: Low back pain; MVC: Maximal voluntary contraction; SD: Standard deviation; TPT: Time to peak torque; TVR: Tonic vibration reflex; VE: Variable error.

## Competing interests

The authors declare that they have no competing interests.

## Authors’ contributions

All authors were equally involved in the conceptualization and design of the study. JAB recruited subjects, managed data collection, completed data analysis and drafted the manuscript. MCN and MD supervised data collection, assisted with drafting and provided critical revision of the manuscript. All authors read and approved the final manuscript.
